# Supplementary figures and images for: The masked seducers: Lek courtship behavior in the wrinkle-faced bat Centurio senex (Phyllostomidae)
Source: PLoS One. 2020 Nov 11;15(11):e0241063. doi: 10.1371/journal.pone.0241063 (PMC7657542; doi:10.1371/journal.pone.0241063)

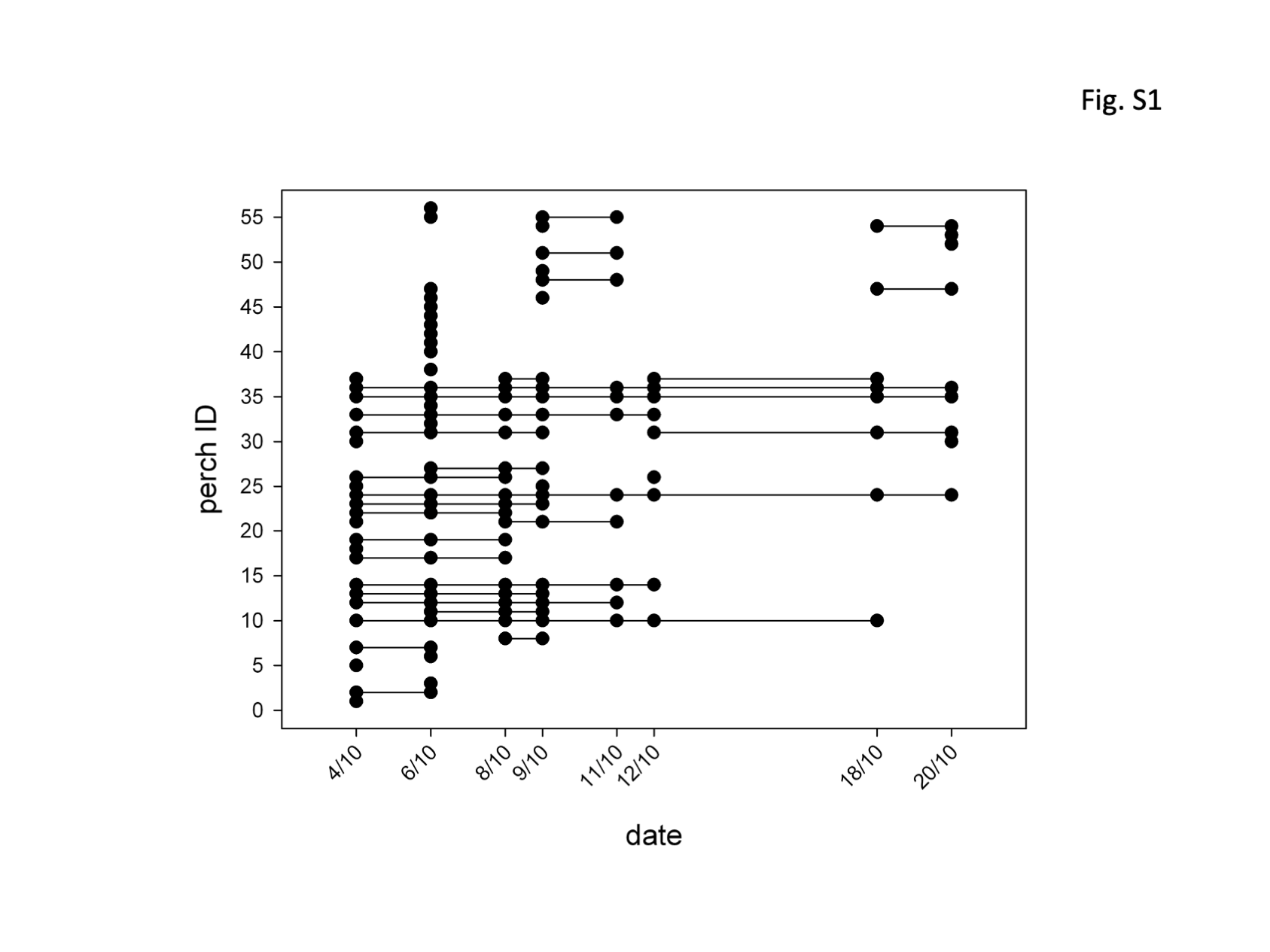

Supplement: S1 Fig — Dots indicate the presence of a Centurio senex male at the respective perch. Perches occupied during successive surveys are connected by a line. (TIFF) [file pone.0241063.s001.tiff]
