# Supplementary material for: The masked seducers: Lek courtship behavior in the wrinkle-faced bat Centurio senex (Phyllostomidae)
Source: PLoS One. 2020 Nov 11;15(11):e0241063. doi: 10.1371/journal.pone.0241063 (PMC7657542; doi:10.1371/journal.pone.0241063)
Supplement: S1 Table — (DOCX) [file pone.0241063.s002.docx]

S1 Table: Comparison between echolocation calls emitted flying and during lek. The echolocation calls are not significantly different for the tested parameters. Wilcoxon rank sum test was used for non-parametric data. For parametric data with equal variances we used Two Sample t-test. If variances were not equal we used Welch Two Sample t-test.

| parameter | test | statistics | df | p-values | Mean flying | Mean lek |
| --- | --- | --- | --- | --- | --- | --- |
|  |  |  |  |  |  |  |
| peak frequency | Wilcoxon rank sum test | W = 10 |  | 0.53 | 91.2 kHz | 88.6 kHz |
| start frequency | Welch Two Sample t-test | t = -0.79 | 7.2 | 0.46 | 112.7 kHz | 116.5 kHz |
| end frequency | Two Sample t-test | t = -0.15 | 9 | 0.88 | 76.1 kHz | 76.47 kHz |
| bandwidth | Two Sample t-test | t = -0.69 | 9 | 0.51 | 36.6 kHz | 40.1 kHz |
| duration | Two Sample t-test | t = -0.03 | 9 | 0.98 | 1.3 ms | 1.3 ms |
| pulse interval | Two Sample t-test | t = 0.22 | 9 | 0.83 | 54.2 ms | 52.0 ms |
| repetition rate | Welch Two Sample t-test | t = -0.69 | 3.3 | 0.54 | 23.1 ms | 26.2 ms |
