# Supplementary material for: The masked seducers: Lek courtship behavior in the wrinkle-faced bat Centurio senex (Phyllostomidae)
Source: PLoS One. 2020 Nov 11;15(11):e0241063. doi: 10.1371/journal.pone.0241063 (PMC7657542; doi:10.1371/journal.pone.0241063)
Supplement: S2 Table — (Wing beat sequence: each syllable comprises a wing beat and its associated wing beat call, if present). n is the number of elements analyzed for each song. (DOCX) [file pone.0241063.s003.docx]

|  | **perch** | **n** | **duration [ms]** | | **# of syllables** | |
| --- | --- | --- | --- | --- | --- | --- |
|  |  |  |  |  |  |  |
| **Echolocation sequence** |  | **15** | **537.0** | **±** **544.33** | **13** | **± 5.1** |
|  | 1 | 1 | 334.5 |  | 9 |  |
|  | 2 | 3 | 607.5 | ± 715.16 | 13 | ± 13.4 |
|  | 3 | 3 | 248.2 | ± 164.87 | 8 | ± 3.5 |
|  | 4 | 3 | 333.4 | ± 239.04 | 7 | ± 2.6 |
|  | 5 | 2 | 612.3 | ± 658.51 | 16 | ± 13.4 |
|  | 6 | 2 | 912.9 | ± 1215.85 | 16 | ± 18.4 |
|  | 7 | 1 | 1102.7 |  | 21 |  |
| **Trill sequence** |  | **15** | **102.1** | **± 19.83** | **26** | **± 2.7** |
|  | 1 | 1 | 111.4 |  | 28 |  |
|  | 2 | 3 | 127.9 | ± 17.20 | 29 | ± 5.0 |
|  | 3 | 3 | 110.9 | ± 12.54 | 30 | ± 3.2 |
|  | 4 | 3 | 88.7 | ± 7.16 | 24 | ± 1.5 |
|  | 5 | 2 | 91.6 | ± 22.43 | 25 | ± 4.9 |
|  | 6 | 2 | 83.0 | ± 1.48 | 24 | ± 0.7 |
|  | 7 | 1 | 88.6 |  | 24 |  |
| **Wing beat sequence** |  | **7** | **504.3** | **± 82.11** | **7** | **± 1.0** |
|  | 1 | 1 | 518.2 |  | 7 |  |
|  | 2 | 1 | 458.0 |  | 7 |  |
|  | 3 | 1 | 461.5 |  | 6 |  |
|  | 4 | 1 | 583.2 |  | 7 |  |
|  | 5 | 1 | 381.5 |  | 5 |  |
|  | 6 | 1 | 626.6 |  | 8 |  |
|  | 7 | 1 | 501.3 |  | 7 |  |
| **Whistle squence** |  | **8** | **78.5** | **± 18.73** | **4** | **± 0.4** |
|  | 1 | 2 | 60.9 | ± 39.50 | 4 | ± 1.0 |
|  | 2 | 1 | 82.4 |  | 4 |  |
|  | 3 | 1 | 89.9 |  | 4 |  |
|  | 4 | 1 | 87.3 |  | 4 |  |
|  | 5 | 1 | 84.2 |  | 4 |  |
|  | 6 | 1 | 80.0 |  | 4 |  |
|  | 7 | 1 | 82.8 |  | 4 |  |

S2 Table: Song elements. Duration and number of syllables. (Wing beat sequence: each syllable comprises a wing beat and its associated wing beat call). n is the number of elements analyzed for each song.
